# Clinical, pathological, and comprehensive molecular analysis of the uterine clear cell carcinoma: a retrospective national study from TMRG and GINECO network

**DOI:** 10.1186/s12967-023-04264-7

**Published:** 2023-06-23

**Authors:** Elsa Nigon, Claudia Lefeuvre-Plesse, Alejandra Martinez, Céline Chauleur, Alain Lortholary, Laure Favier, Anne-Sophie Bats, Arnaud Guille, José AdélaÏde, Pascal Finetti, Victoire de Casteljac, Magali Provansal, Emilie Mamessier, François Bertucci, Isabelle Ray-Coquard, Renaud Sabatier

**Affiliations:** 1 Department of Medical Oncology, Aix-Marseille Univ, Inserm, CNRS, Institut Paoli-Calmettes, 232 Boulevard Sainte Marguerite, Marseille, France; 2 Department of Medical Oncology, Centre Eugène Marquis, Rennes, France; 3 Department of Surgical Oncology, Institut Universitaire du Cancer Toulouse Oncopole, Toulouse, France; 4 Department of Medical Oncology, Institut de Cancérologie de la Loire, Saint Etienne, France; 5 Hôpital privé du Confluent, Institut de Cancérologie Catherine de Sienne, Nantes, France; 6 Department of Medical Oncology, Centre Georges-François Leclerc, Dijon, France; 7 Department of Surgical Oncology, Hôpital Européen Georges Pompidou, Assistance Publique Hôpitaux de Paris, Paris, France; 8 CRCM, Predictive Oncology laboratory, Aix-Marseille Univ, Inserm, CNRS, Institut Paoli-Calmettes, Marseille, France; 9 Department of Medical Oncology, Centre Léon Bérard, University Claude Bernard Lyon 1, Lyon, France

**Keywords:** Uterine cancer, Clear cell carcinoma, Tissue micro-array, Genomics, Gene expression profiling

## Abstract

**Background:**

Uterine clear cell carcinomas (CCC) represent less than 5% of uterine cancers. Their biological characteristics and clinical management remain uncertain. A multicenter study to explore both clinical and molecular features of these rare tumors was conducted.

**Methods:**

This multicenter retrospective national study was performed within the French TMRG (Rare Gynecologic Malignant Tumors) network. Clinical data and, when available, FFPE blocks were collected. Clinical features, treatments, and outcome (progression-free survival (PFS) and overall survival (OS)) were analyzed and correlated to the protein (tissue micro-array), RNA (Nanostring nCounter^®^ technology), and DNA (array-Comparative Genomic hybridization and target-next generation sequencing) levels using the tumor samples available.

**Results:**

Sixty-eight patients with uterine CCC were enrolled, 61 from endometrial localization and 5 with cervix localization. Median age at diagnosis was 68.9 years old (range 19–89.7). Most tumors were diagnosed at an early stage (78% FIGO stage I–II). Hysterectomy (performed in 90%) and lymph node dissection (80%) were the most frequent surgical treatment. More than 70% of patients received external beam radiotherapy and 57% received brachytherapy. Nearly half (46%) of the patients received chemotherapy. After a median follow-up of 24.7 months, median PFS was 64.8 months (95 CI [5.3–124.4]) and median OS was 79.7 (IC95 [31.0–128.4]). Low hormone receptor expression (13% estrogen-receptor positive), frequent PI3K pathway alterations (58% PTEN loss, 50% *PIK3CA* mutations), and P53 abnormalities (41%) were observed. Mismatch repair deficiency was identified in 20%. P16 expression was associated with shorter PFS (HR = 5.88, 95 CI [1.56–25], *p* = 0.009). Transcriptomic analyzes revealed a specific transcriptomic profile notably with a high expression of immune response-associated genes in uterine CCC displaying a very good overall prognosis.

**Conclusions:**

Uterine CCC reported to be potentially MSI high, hormone receptors negative, and sometimes *TP53* mutated. However, some patients with immune response-associated features and better prognosis may be candidate to treatment de-escalation and immunotherapy.

**Supplementary Information:**

The online version contains supplementary material available at 10.1186/s12967-023-04264-7.

## Background

Uterine clear cell carcinomas (CCC), arising both from the endometrium and the cervix are rare gynecological malignant tumors, representing less than 5% of all uterine epithelial cancers [1, 2]. Only few data were published concerning the clinical specificities of uterine CCC. Most of them are described in small subgroups from prospective studies focused on endometrial or cervix carcinoma taken as a whole, or in retrospective cohorts [3, 4]. Molecular descriptions of these rare diseases are also scarce [5]. Even though preliminary data have been published suggesting that endometrial CCC can be closer to ovarian CCC than to endometrial endometrioid tumors [6, 7], most of uterine CCC pathogenesis remains unknown. Therapeutics guidelines are also not specific for this pathological type and are based on guidelines designed for more frequent types: endometrial endometrioid carcinoma and cervical squamous cell carcinoma [8]. Description of more robust data related to these cancers is thus warranted to improve our knowledge and the clinical management. The TMRG (*Tumeurs Malignes Rares Gynecologiques*) network is a national French National Cancer Institute-accredited network dedicated to rare gynecological malignant tumors management including systematic second lecture for pathology and dedicated regional and national tumor boards. One of the main goal is also to develop multicenter research projects focused on rare gynecological cancers [9–11].

As first objective, we aimed to describe clinical presentation at diagnosis and treatment-related data of all patients with uterine clear cell cancer recorded in the TMRG network. Secondary objectives were to describe survival data (Progression-Free Survival and Overall Survival), as well as protein, transcriptomic, and genomic profiles and to evaluate their prognostic value.

## Patients and methods

This work was a retrospective, multi-center, observational study. We retrieved all cases recorded in the TMRG database with sufficient pathological and clinical data available with a data cut-off on November 2017. Patients enrolled in this study should have been diagnosed with endometrium or cervix carcinoma with a clear cell component representing at least 10% of the tumor. For biological analyzes, they should have personally signed and dated informed consent (whether TRMG network or institutional consents). Patients with non-uterine CCC or with uterine carcinoma without CC component were excluded. All tumor samples were reviewed by expert pathologists as systematically performed within the TMRG network.

Our major endpoint was to describe patients (age, body mass index, hypertension, diabetes, in utero exposition to diethylstilbestrol, and personal and family history of cancer) and disease (FIGO stage, lymph node involvement, lymphovascular invasion, and pure clear cell or mixed histology) features. We have also collected treatment-related data: surgical procedure details (pathological margins status, lymph node dissection), external radiation therapy or brachytherapy data (dose (grays) and number of fractions), administration of chemotherapy (drugs identification, number of chemotherapy courses, sequential or concomitant to radiation therapy).

### Immunohistochemistry

For cases with FFPE samples available, we analyzed the expression of proteins of interest by immunohistochemistry (IHC). Experiments were performed using a Tissue MicroArray (TMA) on which each case was deposited in duplicate with cores of 1 mm of diameter. We assessed the expression of ER (estrogen receptor, EP1 clone–Dako/Agilent), PR (progesterone receptor, PgR 636 clone–Dako/Agilent), P53 (DO7–Dako/Agilent), PTEN (6H2.1, Dako/Agilent), P16 (E6-H4, CINtec), PDL1 (22C3 pharmDx–Dako Omnis), mismatch repair proteins (MLH1 (ES05–Dako/Agilent), PMS2 (EP51–Dako/Agilent), MHS2 (FE11–Dako/Agilent), MSH6 (EP49–Dako/Agilent)), and EZH2 (D2C9–Cell Signaling). All stainings were performed using the Dako Link or Dako Omnis (for PDL1) autostainers (Agilent technologies™) with antibodies used at ready to use concentration, except for P53, PTEN, and EZH2 for which antibodies were diluted at 1/100, 1/50, and 1/2000, respectively. Antibodies staining were incubated for 20 to 40 min and revealed with the EnVision Flex kit (Agilent technologies™), according to manufacturer’s instructions. Mouse linkers were used for PgR, PTEN, PDL1, MLH1, and MSH2. A rabbit linker was used for PMS2. The threshold for positivity was set at 10% for hormone receptors, 10% for mismatch repair proteins, 10% for PTEN, and 80% for P53. As no specific data was available for clear cell uterine carcinoma, these thresholds were defined according to data validated in other disease localizations or other pathological subtypes [12–17]. Cases with no P53 staining, i.e. 0%, were also considered as mutant in comprehensive prognostic analyzes if the gene sequencing confirmed the mutation status. No a priori threshold was defined for the other markers. We used 28 high-grade endometrioid uterine tumors and 8 ovarian clear-cell tumors as controls to differentiate organ-related specificities to abnormalities associated with the clear-cell histology. These control samples were explored within the same TMA experiments beside clear cell cases.

### Transcriptomic analyzes

For cases with sufficient tumor area, RNA was isolated and RNA templates were analyzed using the NanoString nCounter^®^ Dx Analysis System [18]. A dedicated custom gene panel was developed including main genes implicated in gynecological cancers. This custom panel included 29 genes plus all genes from the Pan-Cancer pathway panel (770 genes from 13 canonical pathways). 200 to 500 ng of total RNA were used as input and sample hybridization was performed according to the manufacturer's instructions. Sample detection and analysis were completed on an nCounter® Digital Analyzer where genes were counted by scanning 555 Fields-of-view (FOV). The 410 endometrial endometrioid tumors from TCGA (out of 560 samples) and the 237 ovarian clear cell cancer (OvCC) from [19, 20] were used as controls, as well as eleven normal ovarian samples from the same external data set [19, 21].

### Array-comparative genomic hybridization (aCGH)

Array-CGH was performed in order to study DNA copy number profile. Tumor DNA was extracted from FFPE blocks or hematoxylin–eosin–safran (HES) slides by automated methods using the EZ-1 tissue kit, QIAGEN™, according to the manufacturer’s instructions. The cases with DNA of sufficient quality (determined on Agilent Bioanalyzer (Agilent Technologies, Massy, France)) were analysed as previously described [22] using high-resolution 4 × 180 K CGH microarrays (SurePrint G3 Human CGH Microarray Kit, Agilent Technologies, Massy, France). Genomic data from the 393 endometrial endometrioid tumors from TCGA were used as control [21].

### Target next-generation sequencing (t-NGS)

Panel-based next generation sequencing was conducted in cases with DNA that passed quality controls. Tumor DNA was sequenced using a home-made panel of genes as previously described [23]. For each tumor sample, a library of all coding exons and intron–exon boundaries of a panel of 794 target genes (Additional file 1: Table S1) was constructed using the SureSelect enrichment system (Agilent Technologies, Santa Clara, CA, USA). Sequencing was carried out using the Illumina NextSeq500 device (San Diego, CA, USA), according to the manufacturer’s instruction at a median depth of 162×.

### Bioinformatics analysis

#### Single nucleotide mutations analyses

Sequence data were aligned to the human genome (UCSC hg19); alignment and variants calling and annotation were processed as previously described [24]. The Tumor mutational burden (TMB) and MSI (MicroSatellite Instability) score were defined as previously described [23]. Mutations were classified as driver or passenger alterations using the Cancer Genome Interpreter algorithm (https://www.cancergenomeinterpreter.org/home).

#### Array-CGH

All probes for aCGH were mapped according to the hg19/NCBI human genome mapping database. Log2ratio were segmented with Circular Binary segmentation (CBS) algorithm. We used two different threshold values (log2 ratio >|0.15| and |0.9|) to distinguish low (gain/loss) from high (amplification/deletion) level copy-number-alterations (CNA), respectively. Percentage of genome altered was calculated as the sum of altered probes divided by the total number of probes. To identify recurrent copy number alterations, we used the Genomic Identification of Significant Targets in Cancer (GISTIC) 2.0 algorithm calculated by multiple random iterations, with an amplification/deletion threshold > 0.9, confidence level 0.90, and a corrected threshold probability q < 0.25. A HRD (Homologous Recombination Deficiency) score, based on losses of heterozygosity, was calculated for each tumor sample from all tested aCGH genes [25].

#### Gene expression profiling (nCounter^®^ platform, Nanostring™, Seattle, WA, USA)

Raw data processing, quality control, and normalization were performed using the nSolver™ 4.0 analysis software. Briefly, data processing of raw counts was done with background subtraction defined by the geometric mean of the eight negative control probes. Next, quality control of samples was checked according manufacturer requirement in the nSolver™ 4.0. Finally, normalization was done with the geometric mean algorithm using the 40 housekeeping and the six positive control probes. Processed data were then log2-transformed prior analysis. Messenger RNA analyzes were conducted with the Cancer Pathway panel plus 29 custom genes (Additional file 2: Table S2). Unsupervised analysis was done using hierarchical clustering using the Cluster program with data median-centered on genes [26], Pearson correlation as similarity metrics and centroid linkage clustering as parameters. Results were displayed using TreeView program [26]. In association with hierarchical classification, we used quality Threshold (qT) clustering to select clusters of gene by specifying minimum correlation and size values. Robustness of clusters was assessed using the R-package pvclust with uncentered Pearson correlation distance, the average agglomerative method and 100 bootstrap replications as parameters to assess the robustness of clusters [27]. The AU (Approximately Unbiased) p-values provided by multiscale bootstrap resampling indicate the robustness of tumor clusters, larger the p-values, more robust the clusters.

#### Gene expression profiling of public data

Our Nanostring UCCC data were completed by three public transcriptomic data sets including the Uterine Corpus Endometrial Carcinoma data set from TCGA, following Illumina RNA-seq processing, normalization, and publication through the UCSC Xena database [28], and the Winterhoff (GSE73614) and Bolton data sets [19, 20]. We merged these datasets by using COMBAT (empirical Bayes) as batch effects removal method [29], included in the inSilicoMerging R/Bioconductor package [30]. When multiple probes mapped to the same GeneID, we retained the one with the highest variance. Accurate normalization of the merged data sets including 786 commons genes was assessed by t-distributed Stochastic Neighbor Embedding (t-SNE). The 410 endometrial endometrioid tumors from TCGA (out of 560 samples) and the 237 OvCC from [19, 20] were selected for further analysis with our 47 UCCC. Eleven normal ovarian samples from TCGA ovarian cancer data set and Winterhoff’s set were also used as controls. Supervised analysis was done using a moderated t-test with empirical Bayes statistic [31] included in the limma R package (version 3.5.2; http://www.cran.r-project.org/). False discovery rate was applied to correct the multiple testing-hypothesis: the significant genes were defined by p < 0.05%, q < 1%, and fold change (FC) superior to |1.5×| [32]. Using the Gene Expression Signature (GES) obtained, each sample was classified using the nearest centroid algorithm. Robustness of classifier was done by tenfold cross validation using 1000 iterations using same parameters as supervised analysis and prediction accuracy was assessed using exact binomial test with the greater one-sided hypothesis. Ontology analysis of the differential gene list was based on the Reactome terms of the Database for Annotation, Visualization and Integrated Discovery (DAVID; david.abcc.ncifcrf.gov/). In order to assess the potential vulnerability or actionability of tumor samples to certain anti-cancer drugs used or in development in endometrial cancer [33], we applied to each dataset separately the following multigene signatures: two signatures predictive for response to immune checkpoint inhibitors (ICI), the T cell-inflamed signature (TIS) and immunologic constant of rejection signature (ICR) [34, 35], and the Rbsig signature predictive for resistance to CDK4/6 inhibitors [36].

All genomic data supporting our results can be found in Additional file 11: Materials S1, Additionnal file 12: Materials S2.

### Statistical analyzes

The Pearson’s Chi2 test (categorical variables) and Wilcoxon test (continuous variables) were used to compare descriptive items. PFS (progression-free survival) was defined as the time from diagnosis to disease relapse, progression, or death from any cause. OS (overall survival) was defined as the time from diagnosis to death from any cause. Cause of death was collected to discriminate cancer-related events to death from other causes. Data concerning patients without disease progression or death at last follow-up were censored. Survival curves were estimated using the Kaplan–Meier method. Follow-up was estimated by the reverse Kaplan–Meier method. The prognostic impact of clinicopathological features was assessed by the Cox regression method in univariate and multivariate analyses and p-values estimated with the Wald test. All statistical tests were two-sided at the 5% level of significance. This work was done according to the Strengthening the Reporting of Observational Studies in Epidemiology criteria [37].

## Results

### Demographics (Table 1)

**Table 1:** Demographics

	Whole population N = 68	IHC subset N = 42
Age, years		
Median	68.9 (19–89.7)	69.7 (19–89.7)
Tumor location		
Cervix	7 (10%)	4 (10%)
Endometrium	61 (90%)	37 (88%)
Missing data		1 (2%)
Personal history of cancer		
Yes	17 (25%)	10 (24%)
No	49 (72%)	32 (76%)
Missing data	2 (3%)	0
Family history of cancer		
Yes	21 (30%)	12 (29%)
No	38 (57%)	26 (62%)
Missing data	9 (13%)	4 (9%)
Hypertension		
No	47 (69%)	27 (64%)
Yes	19 (28%)	15 (36%)
Absence de donnée	2 (3%)	0
Diabetes		
No	51 (75%)	33 (79%)
Yes	11 (16%)	6 (14%)
Missing data	6 (9%)	3 (7%)
Body Max Index (Kg/m^2^))		
< 18	6 (9%)	2 (5%)
18–25	19 (27%)	12 (28%)
25–30	21 (31%)	15 (36%)
> 30	10 (15%)	7 (17%)
Missing data	12 (18%)	6 (14%)
FIGO stage at diagnosis		
I	38 (56%) Including 16 IA	26 (62%) including 9 IA
II	4 (6%)	2 (5%)
III	15 (22%)	8 (19%)
IV	7 (10%)	5 (12%)
Missing data	4 (6%)	1 (2%)
Lymph node status at diagnosis		
pN0	51 (75%)	33 (78%)
pN1	15 (22%)	7 (17%)
Missing data	2 (3%)	2 (5%)
Lymphovascular invasion		
Yes	29 (43%)	19 (45%)
No	24 (35%)	16 (38%)
Missing data	15 (22%)	7 (17%)

**Fig. 1:**
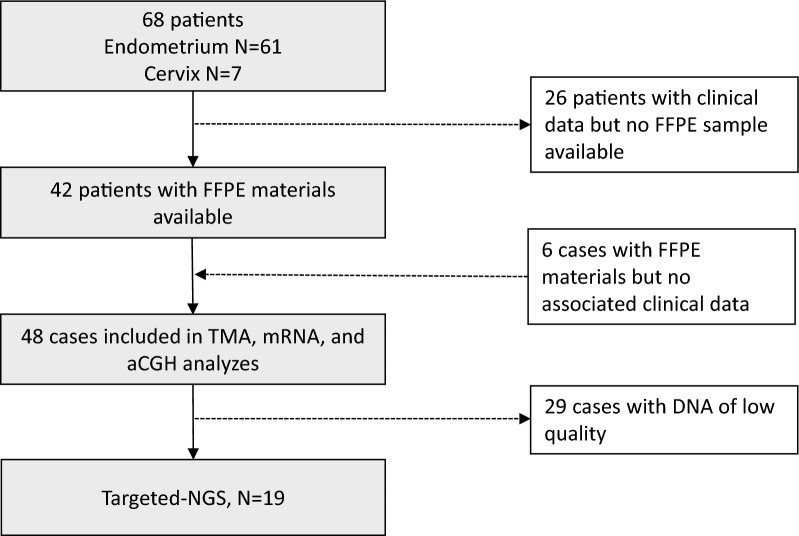
Study flow chart. FFPE: formalin-fixed paraffin-embedded; TMA: tissue micro-array; aCGH: array-based comparative genomic hybridization; NGS: next-generation sequencing

We retrospectively collected 68 cases of uterine cancer with a clear cell component in eight French cancer centers. Patients were diagnosed with uterine cancer from October 2000 until November 2017 (Fig. 1—Flow chart).

Most of them (N = 61, 90%) were diagnosed with endometrium cancer (Table 1). Median age was 68.9 years old (range 19–89.7). Median body mass index was 25.4 kg/m^2^ (range 16.6–50.8) and a minority reported previous history of diabetes (16%) or hypertension (27%). A quarter of them had a personal history of cancer, with breast cancer as the most frequent (88%) previous malignant disease. A third (30%) had a family history (parents, siblings, children) of cancer, with 16 cases of breast cancers, four of colo-rectal cancers, and one of ovarian cancer. We aimed to explore in utero exposition to diethylstilbestrol. Unfortunately, these data was unknown for most patients included in this study and could not be described. Thirty-eight (56%) were FIGO stage I at diagnosis, four (6%) were stage II, 15 (22%) stage III, and seven (10%) stage IV. Fifteen (22%) patients had loco-regional lymph node metastases. Lymphovascular invasion was observed in 29 (43%) cases.

### Treatment

As endometrial cancer represented 90% of our set, we will describe below only treatments received by these patient (details for cervix tumors in Table 2). More than 90% of the patients underwent frontline surgery and forty-four (72%) received external beam radiotherapy, with a dose-equivalent of at least 45 grays in 25 fractions. Thirty-five patients (57%) also received high-dose brachytherapy. Twenty-eight patients (46%) received chemotherapy, mainly based on carboplatin-paclitaxel doublet. The median number of chemotherapy cycles was four (range 1–6).

**Table 2:** Details of treatments received (N = 68)

	**Endometrium** **N = 61**	**Cervix** **N = 7**
Surgery		
Frontline	56 (92%)	5 (71%)
Free margins	50 (89%)	3 (60%)
Pelvic LND	49 (88%)	4 (80%)
Para-aortic LND	40 (71%)	5 (100%)
Radiotherapy		
Radiotherapy alone	26 (43%)	1 (14%)
Radio-chemotherapy (concomitant or sequential)	18 (30%)	3 (43%)
Brachytherapy	35 (57%)	4 (57%)
Chemotherapy		
Carboplatin-paclitaxel	28 (46%)	3 (43%)
Carboplatin-paclitaxel-bevacizumab	21 (75%)	2 (29%)
CAP	1 (4%)	1 (14%)
Carboplatin monotherapy	2 (7%)	0
Carboplatin-adriamycin	2 (7%)	0
Cisplatin monotherapy	1 (4%)	0

LND, lymph node dissection; CAP, cyclophosphamide, Adriamycin, platin

### Clinical outcome

Median follow-up was 24.7 months (95 CI [16.0–33.3]). Among the 68 patients included in this study, 39 (57%) were still disease-free at time of last news. Seventeen (25%) patients experienced disease progression with distant metastases. Ten had a loco-regional relapse prior to or concomitantly with distant metastases. Four other patients displayed pelvic relapse without metastasis. Metastases occurred in lymph nodes (N = 7), lungs or liver (N = 5 each), peritoneum (N = 3), pleura (N = 2), bones (N = 1), and brain (N = 1). Metastatic site details were not available in one patient.

At the date of the database locked, 17 patients were deceased, including 16 for whom death was related to cancer. Out of 51 alive patients, 39 were disease-free, one with disease in partial response to treatments, four with disease progression, and data was missing in seven cases. Five patients were diagnosed with a new primary malignancy during follow-up (three breast cancers, one colorectal cancer, and one kidney cancer).The 2-year PFS and OS rates were 73.1% and 84.5%, respectively (Additional file 6: Fig. S1).

### Analysis of protein expression using immunohistochemistry

Immunohistochemistry (IHC) was performed in 48 patients (71%) for whom FFPE material was available and deposited onto a TMA. Clinical data were available in 42 of them. Clinical features and treatments of this subset were similar to that of the whole population (Table 1). Median follow-up was 20.4 months (95 CI [16.4–24.4]) in this subset, with similar 2y-PFS and OS rates than the whole cohort (65.6% and 78.4%, respectively). Out of the 48 cases, six (12%) were ER-positive and four (8%) were PR-positive, including two ER + /PR + cases (Table 3). By contrast, none of the eight ovarian clear cell controls *(p* = 0.65, Chi2-test for comparison *versus* uterine CCC) and 60% of 29 endometrioid uterine cancer controls (*p* = 1.2 E-05) were ER + . PR expression was also lower in uterine CCC *versus* endometrioid tumors (8% *vs* 58% *p* = 4.8 E−05, and 0% in OvCC, *p* = 0.9). Eleven cases (23%) were P53mut, including three of the five cervix cancers. Nineteen of the 48 cases (40%) presented a loss of at least one MMR (mismatch repair) protein and ten patients lost two markers (N = 7 for PMS2/MLH1, N = 2 for MSH6/MSH2, one tumor lost expression of all four proteins). PTEN was lost in 28 (58%) cases. Only four tumors were PDL1 + (8%), including one with PDL1 expression in more than 50% of tumor cells. We observed P16 expression in at least 10% of tumor cells in 38% of cases. Of the 19 cases with positive EZH2 expression, more than 50% of tumor cells were positive in 12 (25%) samples.

**Table 3:** Protein expression in uterine clear cell cancers (UCCC, N = 48), *versus* uterine endometrial (UEnd, N = 28) and ovarian clear cell (OvCC, N = 8) cases

		N, %		p-value
ER positive	**UCC**	**6**	**13%**	
	**UEnd**	**18**	**64%**	**1.2 E−05**
	OvCC	0	0%	0.65
PR positive	**UCC**	**4**	**8%**	
	**UEnd**	**15**	**54%**	**4.8 E−05**
	OvCC	0	0%	0.90
P16 positive	UCC	18	38%	
	UEnd	14	50%	0.41
	OvCC	4	50%	0.78
P53 mutant profile	UCC	11	23%	
	UEnd	6	21%	0.94
	OvCC	1	13%	0.75
PTEN loss	UCC	28	58%	
	UEnd	18	64%	0.79
	OvCC	4	50%	0.96
PDL1 positive	UCC	4	8%	
	UEnd	0	0%	0.30
	OvCC	0	0%	0.92
MMRd	UCC	19	40%	
	UEnd	9	32%	0.69
	OvCC	0	0%	0.07
MMRd2	UCC	10	21%	
	UEnd	6	21%	1
	OvCC	0	0%	0.36
		Mean (SD)		
EZH2 expression^a^	UCC	24.3% (39.2)		0.38, Kruskal–Wallis
	UEnd	36.6% (41.7)		
	OvCC	17.5% (26.0)		
γH2AX expression^a^	UCC	18.3% (31.1)		0.54, Kruskal–Wallis
	UEnd	24.7% (38.1)		
	OvCC	11.4% (29.8)		

*ER* estrogen receptor; *PR* progesterone receptor; *MMRd* loss of at least one mismatch repair protein; *MMRd2* loss of at least two MMR proteins and considered as MMR deficient; *SD* standard deviation

UCC considered as reference for statistical analyzes

P-values: Chi2-test unless specified

Features with significant differences are in bold

^a^Percentage of positive cells

Except for hormone receptors expression, rates of positive tumors were similar between UCCC and endometrial endometrioid tumors. For instance, no significant difference was observed for MMR proteins taken alone (*p* = 0.69) or in dual combination (MLH1/PMS2 or MSH2/MSH6; *p* = 1). None of the ovarian CCC controls was negative for MMR proteins.

### Analysis of gene expression profiles

**Fig. 2:**
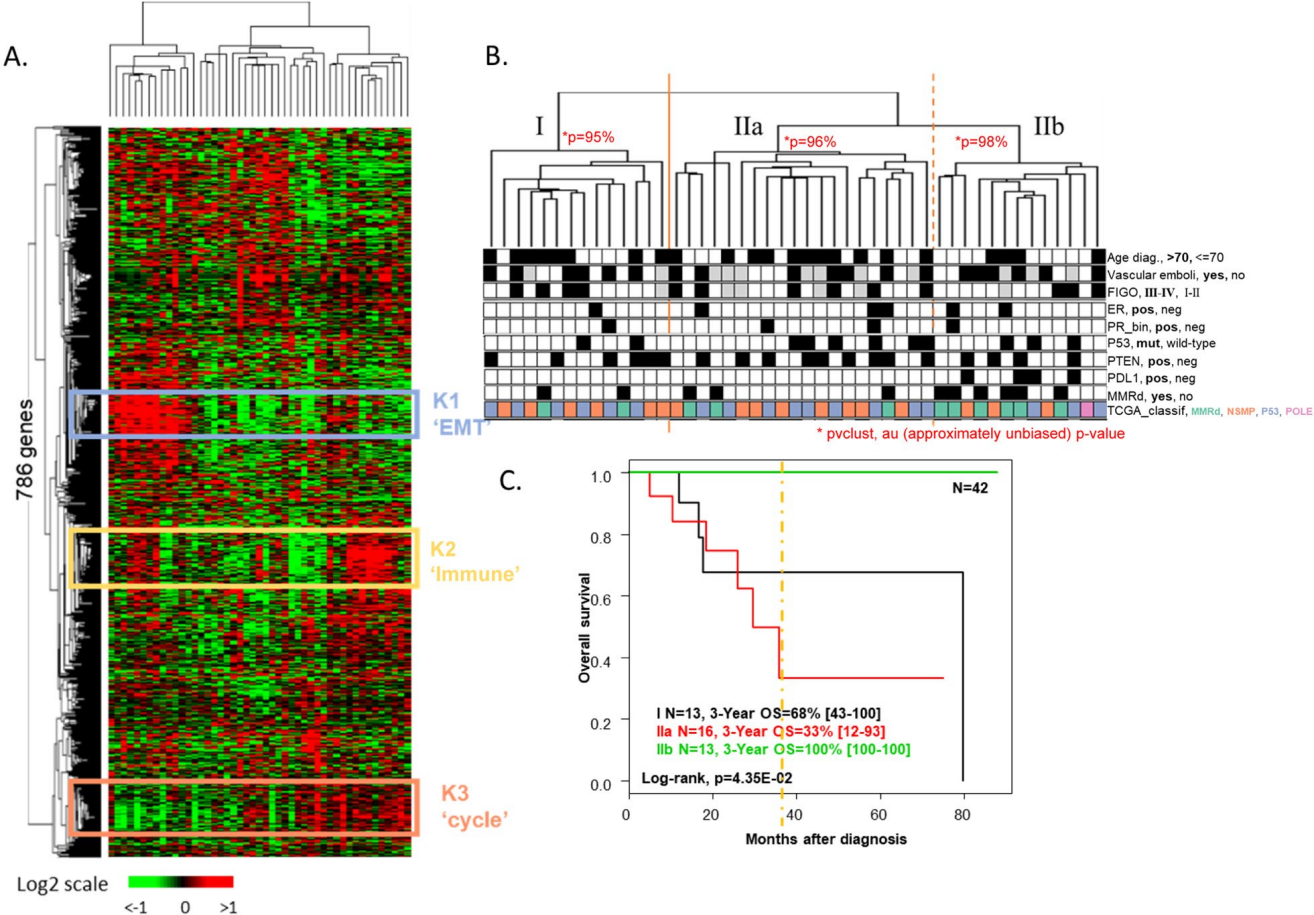
Gene expression profile of uterine CCC according to the 786-gene panel. **A** Hierarchical clustering (N = 47) with main three quality threshold clusters with Pearson correlation > 0.6 and including at least 20 genes defined by expression of genes related to epithelial-mesenchymal transition (EMT), immune response, and cell cycle. **B** Correlation with main clinical and pathological data. Pvclust R-package was used to explore clusters robustness, with approximately unbiased *p*-values. **C** Kaplan Meier curves of overall survival

Unsupervised transcriptomic analysis of 47 tumors identified three subgroups with similar clinical features. Tumors from the cluster I were characterized by high expression of genes involved in epithelial-mesenchymal transition (EMT). Tumors from a second cluster (IIb) had high expression of immune response and cell cycle-related genes (Fig. 2A). Most tumors classified as MMRd by IHC, the *POLE*-mutated tumor, and all cases with PDL1 expression belonged to this cluster (Fig. 2B). The third tumor cluster (cluster IIa) was characterized by a lower expression of genes associated with EMT and immune response.

**Fig. 3:**
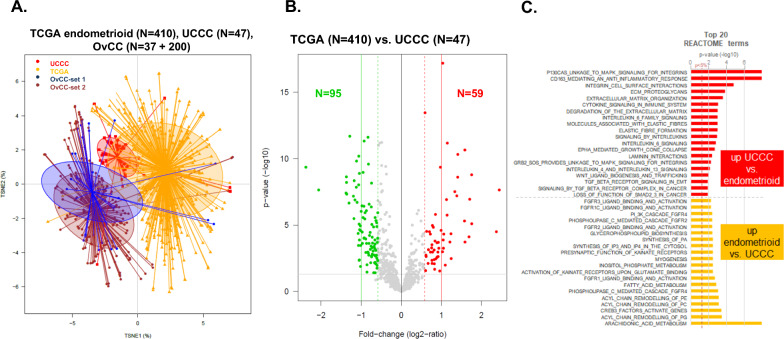
Analysis of gene expression profiles of uterine CCC, endometrioid tumors from TCGA, and ovarian clear cells (OvCC) tumors [19, 20]. **A** t-SNE (t-distributed Stochastic Neighbor Embedding) unsupervised analysis based on all 786 genes showing that centroid of the UCCC set is closer to all endometrial carcinoma than to ovarian clear cell carcinoma. **B** Volcano plot of differential mRNA expression between uterine CCC and the TCGA endometrioid endometrial carcinoma data set with 154 genes differentially expressed (*moderated t-test: p* < *5%, q* < *10% *and* |FC|*> *1.5x*). **C** Ontology analysis based on the Reactome database. The best 20 pathways are represented

We then included the control tumor samples. Unsupervised analysis showed that uterine CCC are closer to endometrioid endometrial cancers than to OvCC (Fig. 3A). Nevertheless, transcriptomic profiles of uterine CCC (N = 47) and endometrioid endometrial cancers (N = 410) were not fully similar and supervised analysis identified 154 genes differentially expressed between them (Fig. 3B). To remove the genes that could be associated to TCGA subtype obtained by ProMisE molecular classification [38], a multivariate logistic regression analysis with the glm function in R’s stastistical package was done on the 154 genes to compare uterine CCC to endometrioid endometrial tumors stratified with TCGA subtype. Off the 154 genes, nine were classified as dependent to the ProMisE classification and excluded from further analysis. Ontology analysis of the remaining 145-gene list showed that genes involved in immune response and cellular matrix regulation were more expressed in uterine CCC than in endometrioid tumors (Fig. 3C, Additional file 3: Table S3). At the opposite, uterine CCC had lower expression of genes associated with fatty acids metabolism and FGF-related pathways.

The number of genes tested (786 genes) did not allow to assess the potential vulnerability (based on gene expression signatures predictive for response or resistance) or actionability (based on gene expression level of the target protein) of tumor samples to most of anti-cancer drugs used or in development in endometrial cancer [33]. That was possible for a few drugs. HER2 expression, potential target of trastuzumab-based antibody–drug conjugates (ADCs), was more expressed in uterine CCC than in TCGA endometrioid endometrial carcinoma (*p* = 1.28E-04). Similarly, two signatures predictive for response to immune checkpoint inhibitors (ICI) showed higher score in uterine CCC: TIS (*p* = 3.90E−05) and ICR (*p* = 9.520E−03) [34, 35], suggesting higher potential vulnerability to ICI in uterine CCC. Conversely, the Rbsig signature showed higher score in uterine CCC (*p* = 0.082), suggesting lesser vulnerability to CDK4/6 inhibitors than in endometrioid endometrial carcinoma [36].

After filtering of organ-specific genes, a similar approach for uterine CCC *versus* OvCC comparison identified 207 differentially expressed genes (Additional file 7: Fig. S2, Additional file 4: Table S4). TRKA receptors and synaptic signals pathways were upregulated in uterine CCC, whereas extracellular matrix cell deaths associated genes were downregulated compared to OvCC.

### Analysis of copy number alterations using aCGH

We performed aCGH on 47 uterine CCC samples. GISTIC-2.0 analysis identified numerous copy number alterations (CNAs). Notably, we observed frequent amplifications in 6p11.2 (*PRIM2*), 8p11.22 (*IKBKB*), 14q24.3 (*SNW1*), and 17q12 (*ERBB2* and *CDK12*), Additional file 8: Fig. S3A). Deletions in regions of interest were also identified: 1p36.32, 6p21.32 (*DAXX*), 8p11.22 (*ADAM* family genes and *TM2D2*), 15q11.2 (*UBE3A*), and 19p13.3 (*STK11*).

Supervised analysis of CNAs identified in our uterine CCC cohort (N = 47) *versus* the TCGA data set (N = 393) showed that several regions were differentially altered (Additional file 8: Fig. S3B). 8p11.21, 10q22.2, 12q13.3, 14q24.3, 17q12, and 17q23.1 regions were statistically more frequently amplified in uterine CCC. Regions more frequently deleted were 1p31.1, 1q21.3–31.3, 4q13.2, 12p13.31, and 20p13.The HRD score was higher in uterine CCC than in TCGA endometrioid tumors (*p* = 2.39E−06, Student t-test; Additional file 8: Fig. S3B), suggesting potential higher vulnerability of CCC to PARP inhibitors.

### Analysis of mutational profiles using t-NGS

**Fig. 4:**
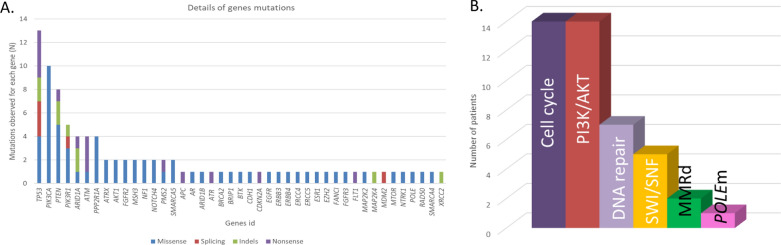
Alterations identified with sequencing-NGS (N = 19). **A** Details of gene mutations classified by genes and types of mutations. **B** Distribution of gene alterations in the main pathways of interest

After DNA quality control assessment, 19 of the 48 cases were available for t-NGS analysis covering 794 target genes. One hundred and ninety-one pathogenic driver alterations were identified in 118 genes (Fig. 4A and Additional file 5: Table S5). Most observed alterations (67%) were missense single nucleotide variations, followed by nonsense mutations (15%), indels 15%) and splicing mutations (3%). All analyzed tumors displayed at least one mutation of interest. Fourteen tumors (74%) had mutations in genes involved in the PI3K/AKT/mTOR pathway (Fig. 4B). Seven tumors had mutations in genes involved in DNA repair pathways. Genes coding for proteins of the SWI/SNF complex were also altered in five cases (26%). We observed mutations in genes coding for MMR proteins in two tumors (11%). *POLE* was mutated in one case. Fourteen (74%) of analyzed samples had alterations in cell cycle-related genes: *TP53* (13 mutations), *PPP2R1A* (four mutations), and *CDKN2A* (one mutation).

### ProMisE molecular classification

We combined results from IHC and t-NGS in order to classify tumors in one the four molecular subtypes identified in the TCGA cohort: *POLE*-mutated, MMR deficient by IHC, P53 mutant (P53 pos by IHC and/or *TP53*-mutated by t-NGS), and cases of non-specific molecular profile (NSMP). We identified one (2%) *POLE*-mutated tumor, 11 (23%) MMRd, 18 (38%) P53 mutant, and 18 (38%) NSMP cases (Additional file 9: Fig. S4A). As expected, we found correlations between these subtypes and genomic scores. The HRD score tended to be higher in the P53mut subtype (*p* = 0.072, Student t-test; Additional file 9: Fig. S4B), the MSI score tended to be higher in the MMRd subtype (*p* = 0.107, Student *t*-test; Additional file 9: Fig. S4C), and the TMB was higher in the *POLE*-mutated subtype (*p* = 0.048, Student *t*-test; Additional file 9: Fig. S4D), suggesting the coherence of data.

### Correlation of clinical, pathological, and genomic alterations to survival (PFS)

Prognostic assessment of clinical features identified lymph node involvement as the only parameter tending to be associated with PFS (HR = 2.32; 95 CI [0.95–5.88]; *p* = 0.06, Wald’s test). No other baseline clinical feature was prognostic (Table 4). Patients who did not received radiotherapy or brachytherapy had a shorter PFS: HR = 3.07 (95 CI [1.26–7.48]) for radiotherapy, and HR = 3.08 (95 CI [1.21–7.86]) for brachytherapy. However, this was due to a lower rate of local treatment in patients with advanced stage disease. Forty-four percent of patients with FIGO stage III and IV tumors received radiotherapy *versus* 83% in FIGO stage I-II (*p* = 0.003, Pearson correlation). This was similar for brachytherapy: 29% *versus* 74%, *p* < *0.001*).

**Table 4:** Cox univariate analysis of progression-free survival including baseline clinical and treatment criteria

Variables		N	HR	95 CI	*p*-value
Age	Continuous	68	1.02	0.97–1.06	0.45
BMI	Continuous	62	1.04	0.97–1.10	0.26
Localization	Cervix vs endometrium	68	1.92	0.56–6.69	0.3
Surgical margins	Pos vs neg	58	2.5	0.77–7.69	0.13
pN	Pos vs neg	66	2.32	0.95–5.88	0.06
LVI	Pos vs neg	53	2.17	0.70–6.67	0.18
FIGO stage	I-II vs III-IV	64	0.53	0.22–1.32	0.17
Chemotherapy	No vs yes	68	0.63	0.21–1.32	0.17
Radiotherapy	No vs yes	68	3.07	1.26–7.48	0.014
Brachytherapy	No vs yes	65	3.08	1.21–7.86	0.019

*BMI* body mass index; *LVI* lymphovascular invasion

**Fig. 5:**
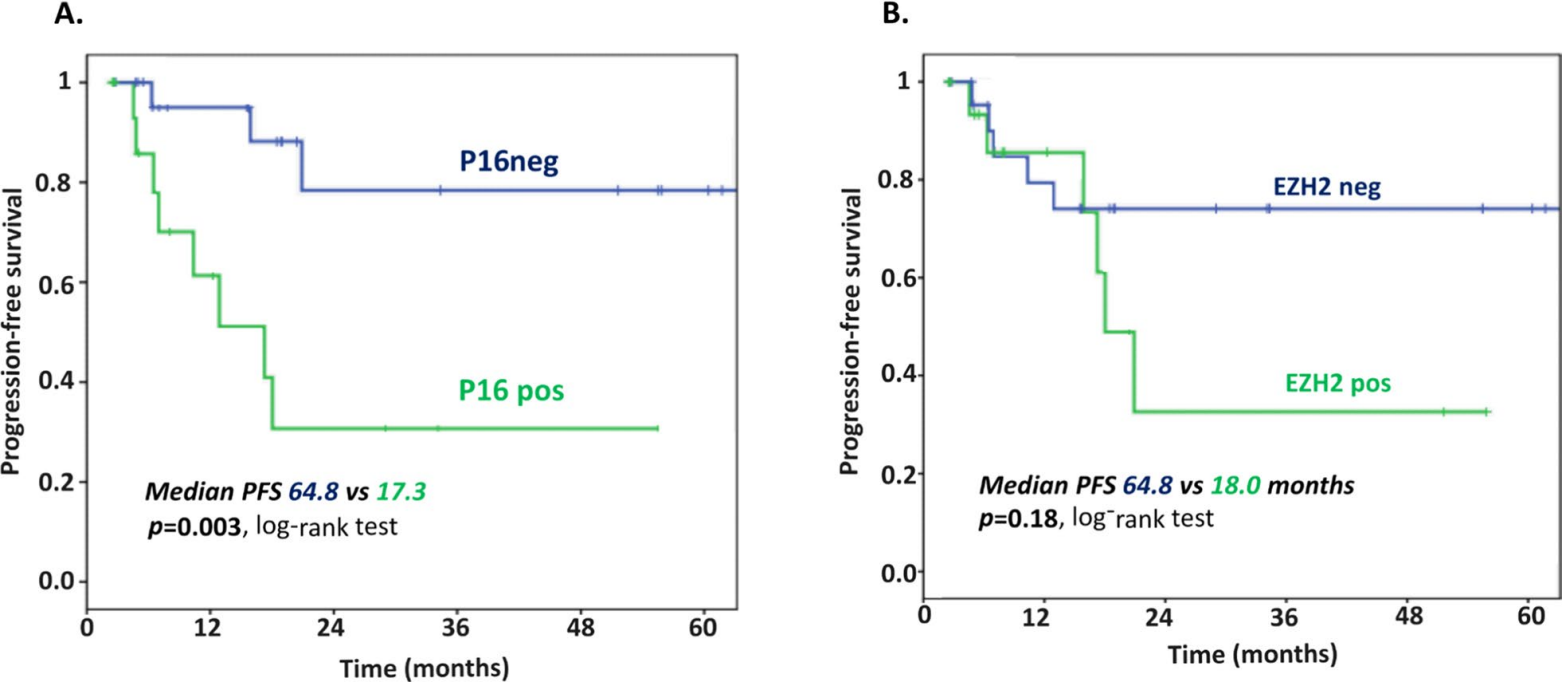
Kaplan Meier curves for progression-free survival according to **A** P16 expression, and **B** EZH2 expression

Univariate analysis identified P16 as the only IHC marker significantly associated with PFS (Table 5). Cases without expression of P16 had a longer median PFS than cases with expression (64.8 months *vs* 17.3 months respectively; *p* = 0.003, log-rank test; Fig. 5A). Multivariate analysis for PFS including p16, FIGO stage (I–II vs. III–IV), and lymphovascular invasion showed that P16 prognostic value was independent from these clinicopathological features: HR = 6.67, 95 CI [1.37–33.33] for P16; HR = 0.70, 95 CI [0.19–2.52] for FIGO stage; HR = 0.99, 95 CI [0.23–4.29] for lymphovascular invasion. P16 Protein and *CDKN2A* (gene coding for P16) mRNA expression were correlated (p = 1.37 E-03, Student’s *T*-test), and *CDKN2A* expression tended to be correlated to PFS (HR = 1.35 [0.98–1.85], *p* = 0.06, Wald’s test, N = 36), reinforcing p16 prognostic value. Median PFS was 18.0 months in EZH2-positive cases *versus* 64.8 months in EZH2-negative tumors, but the small sample size could not lead to significance (*p* = 0.18, log-rank test, Fig. 5B).

**Table 5:** Cox univariate analysis of progression-free survival including IHC markers, Wald's test

Variables		N	HR	95 CI	*p*-value
ER	Pos vs neg	41	1.39	0.36–5.26	0.63
PR	Pos vs neg	41	0.81	0.10–6.25	0.84
GH2AX	Continuous	41	0.99	0.98–1.02	0.97
EZH2	Continuous	41	1.01	0.99–1.03	0.09
P16	Pos vs neg	41	5.88	1.56–25	0.009
P53	Pos vs neg	41	0.58	0.13–2.70	0.49
PTEN	Pos vs neg	41	1.75	0.53–5.88	0.35
PDL1	Pos vs neg	41	0.04	0–1000	0.55
MMRd	Yes vs no	41	1.01	0.27–3.84	0.99

The prognostic impact of the ProMisE classification in this clear cell tumors cohort was then explored. No survival difference between MMRd, P53mut, and NSMP subgroups (*p* = 0.97, log-rank test, Additional file 9: Fig. S4E) was observed. Nevertheless, patients in the “immune response subgroup” of transcriptomic analyzes experienced a very good prognosis, with a 3-y overall survival of 100% *versus* 33 to 68% in the two other clusters (Fig. 2C). Moreover, to reinforce the robustness of this classification in the absence of external validation set, we used the Pvclust algorithm and confirmed the low uncertainty of the hierarchical clustering with AU p-values of 95% to 98% [27].

Supervised analysis of aCGH data using GISTIC2.0 algorithm did not identify copy number alterations correlated to disease progression at the False Discovery Rate threshold of 0.25 (Additional file 10: Fig. S5). Alterations of the PI3K/AKT, MAPK, DNA repair, or SWI/SNF pathways, explored by TMA, NGS, and aCGH, were not predictive of poorer PFS: HR = 2.38 [0.62–9.10] for PI3K, HR = 1.09 [0.29–4.13] for MAPK, HR = 1.86 [0.50–6.92] for DNA repair, and HR = 0.52 [0.15–1.79] for SWI/SNF.

## Discussion

This national retrospective study focused on a rare tumor type reports similar clinical features between uterine clear cell carcinoma and uterine endometrioid cancer. However, they have different biological profiles with specific molecular profiles. Comprehensive pathological, transcriptomic and genomic analyzes identified P16 and expression of immune response-related genes as potential prognostic markers in this subtype (Table 6).

**Table 6:** Summary of most relevant findings

	IHC	aGCH	Gene expression
Main alterations in UCCC vs endometroid cancer	Lower ER expression (13% vs 64%)	Amplifications of 10q22.2 (*ADK*), 14q24.3 (*SNW3*), 17q12 (*ERBB2*), 17q23.1 (*VMP1*)	Increase expression of genes related to immune response and extra-cellular matrix
	Lower PR expression (8% vs 54%)	Deletions of 4q13.2 (*UGT2B17*), 20p13 (*SIRPB1*)	Decrease expression of genes associated with fatty acid metabolism and FGF-related pathways
		Higher HRD score	
Prognostic alterations	P16 expression	None	Immune cluster based on mRNA unsupervised analysis
	HR = 5.88; 95 CI [1.56–25]		*p* = 4.35 E−02, log rank test

*IHC* immunohistochemistry; *aCGH* array-comparative genomic hybridization; *UCCC* uterine clear cell carcinoma; *ER* estrogen receptor; *PR* progesteron receptor; *HRD* homologouis recombination deficiency; *HR* hazard ratio; *95 CI* 95% confidence interval

If median age at diagnosis among the 68 patients enrolled in this cohort (69 years old) was similar to that of endometrioid uterine cancer [39], the frequencies of prior history of hypertension and diabetes were similar to general population, whereas they are more frequent in patients with endometrioid tumors. Consistently with literature, most patients were diagnosed with early stage disease (FIGO stage I in 56%, stage II in 22%). As supported by surgical guidelines in non-endometrioid tumors, most patients underwent surgery with lymph node dissection and received adjuvant radiotherapy. Chemotherapy administration was less frequent (< 50%) despite an aggressive histology classified as high-risk even when diagnosed at stage I [40].

Patients enrolled in this study have a good short-term outcome, with 2y-PFS and 2y-OS rates of 73.1% and 84.5%, respectively. Previous observations have already highlighted than stage I-II UCCC have a median survival similar to that of endometrioid uterine tumors and that UCCC have better prognosis than serous uterine tumors [1, 41]. We identified p16 loss of expression as the main feature associated with survival. Lower expression of p16 protein and under-expression of *CDKN2A* (gene coding for p16) were associated with increased PFS. To our knowledge, it is the first report of p16 prognostic value in uterine CCC. However, this has already been described in ovarian CCC with patients with no p16 expression displaying longer OS [42]. Endometrioid endometrial tumors with p16 overexpression also have a poorer prognosis [43].

Recent guidelines recommend that adjuvant treatments for endometrial cancer should be based on the ProMisE classification [38]. The cancer genome atlas classification has identified four molecular subgroups (p53-abnormal, *POLE*-mutated, MMRd/MSI-high, and tumor of non-specific molecular profile) [21]. This classification has a prognostic impact in endometrioid and serous subtypes but its correlation to outcome in rare pathological subtypes remains unclear. In a small-size single institution uterine CCC cohort, 2% of tumors were *POLE*-mut, 10% MMRd, 54% NSMP, and 35% p53abn [44]. This is close to what we observed in our cohort: 2% *POLE*-mut, 23% MMRd, 38% p53-abn, and 38% NSMP. We may have an increase rate of MMRd tumors as our set included mixed tumors that have been associated with higher rates of MMR alterations than pure CCC [45]. This is of interest as these patients may have a better outcome than MMR proficient CCC [46]. The limited sample size of our set may have precluded us to show a prognostic value of the ProMisE classification in uterine CCC. However, other small size cohorts also failed to demonstrate significant prognostic value of this classification, even though p53-abnormal cases seem to display the poorest outcome [44].

Nevertheless, transcriptomic analyzes showed that overexpression of genes associated with immune response was correlated to *POLE*-mut and MMRd subtypes, and associated with improved survival. Therefore, gene expression data may be a more efficient prognostic marker than the ProMisE classification for the UCCC population. However, due to our limited sample size, validation using an independent data set is warranted to confirm these results before suggesting that patients with an immune mRNA profile may benefit from treatment de-escalation. Gene expression analysis also showed that uterine CCC are closer to uterine endometrioid tumors than to ovarian clear cell carcinoma. This may limit the development of tumor agnostic clinical trials only based on pathological types.

Our genomic analyzes identified other altered pathways. Alterations of the PI3K/AKT pathway (*PI3KCA, PTEN*, or *AKT1* mutations, *STK11* deletions, PTEN loss) were frequent, as well as DNA repair (*ATM, ATRX, ATR, BRCA1*) and SWI/SNF complex (*ARID1A, ARID1B, EZH2, SMARCA4*) alterations. PI3K/AKT/mTOR pathway alterations are frequent in CCC, with more than one third with *PI3KCA* mutations and 40% with PTEN loss [47, 48]. PI3K, AKT, and mTOR inhibitors are under investigation in endometrial cancer with promising results in early phase studies [49–51]. DNA repair alterations (whether HRD-related genes mutations and/or high genomic instability score) we observed in our set may also be a target. PARP inhibitors are explored in the recurrent setting [52]. SWI/SNF pathway alterations are frequent in endometriosis-related tumors, including clear cell and endometrioid ovarian carcinoma [20, 53, 54]. Recent circulating tumor DNA data suggest that endometrial cancer is the tumor localization with the highest *ARID1A* mutation rate (21%). This may offer new opportunities for endometrial cancer treatment with the assessment of EZH2 inhibitors or ATR inhibitors in the setting of recurrent uterine CCC [55, 56]. Finally, we identified frequent 17q12 (*ERBB2* amplicon) amplification. As some preliminary data suggest ERBB2-targeting efficacy in ERBB2-positive endometrial serous cancer and in a few case reports of uterine CCC [57, 58], use of trastuzumab or trastuzumab-based ADCs warrants to be further explored in *ERBB2*-amplified uterine CCC.

Despite these important findings, our study presents some limitations as sample size may have limited the power of our study and our capacity to identify some prognostic features, especially to perform multivariate analyses including genomic and transcriptomic classifications. However, as uterine CCC is a rare disease; comprehensive molecular studies in larger sets are difficult to conduct outside national and international networks such as the TMRG network. Moreover, the TMRG and the GINECO group have shown their expertise in rare gynecological tumors management by sponsoring randomized clinical trials in rare diseases such as sex cord-stromal tumors [59]. The BOUQUET study (NCT04931342), a basket trial dedicated to rare ovarian tumors is ongoing. A similar design may be developed for advanced endometrial cancer with specific molecular alterations. One of the potential limitation was to include both pure and mixed CCC, partially guided by real-world practice where CCC uterine tumors often display other components (mainly endometrioid or serous). For instance a recent cohort reported 60% of mixed uterine CCC [60]. Third, our protein analyzes were based on TMA results. Tumor heterogeneity may thus have been missed. Concerning transcriptomic analyzes, no external validation could be done as no independent public data is available in uterine CCC. Finally, the low amount of DNA of sufficient quality (old FFPE samples) did not allow us to explore mutation profile in all cases. Further NGS data would have been of interest.

In conclusions, we show with this comprehensive clinical, pathological and molecular study that such effort can be conducted in rare gynecological disease by national networks. This reinforces the need of collaborations and molecular profiling to identify potentially actionable alterations of such diseases and our capacity to propose individualized therapies based on ESCAT (ESMO Scale for Clinical Actionability of molecular Targets) evaluation [61]. Our data may help to propose new therapeutic alternatives to our patients and to develop new prospective trials in uterine clear cell carcinoma.

## Supplementary Information

**Additional file 1: Table S1.** V11 NGS panel.

**Additional file 2: Table S2.** Genes added to the Cancer Pathway panel for transcriptomic analyses.

**Additional file 3: Table S3.** Supervised Analysis, 410 Endometrioid Endometrial Uterine Cancer (EEUC from TCGA) vs. 47 UCCC.

**Additional file 4: Table S4.** Supervised analyses, 237 celar cell ovarian cancers (OvCC) vs. 47 UCCC.

**Additional file 5: Table S5.** Deleterious DNA mutations identified by NGS (N=16 UCCC)

**Additional file 6: Figure S1.** Kaplan Meier curves for progression-free survivaland overall survival.

**Additional file 7: Figure S2.** Supervised analysis of gene expression profiles of UCCC and a set of ovarian clear cells tumors.Volcano plots of differential mRNA expression between UCCC and ovarian clear cell tumorsand normal ovarian tissue. Filtering of ovarian tissue related genes allow idenitification of 207 differentially expressed genes.

**Additional file 8: Figure S3.** Copy number alterations profiles of uterine CCC tumors from the TMRGcohort.Frequency plot of recurrent copy number alterations identified in UCCC tumors using the GISTIC algorithm. Frequencies of gainsand lossesare plotted as a result of chromosome location. X-axis: top = log–scale ratio; bottom = q-values. Green lines represent the threshold for significance.Supervised analysis comparing uterine CCC samples to endometrial tumors from TCGA dataset. Dotted line: Threshold of significance associated with a False Discovery Rate < 0.25.Homologous recombination deficiencyscore between TMRG and TCGA cohorts.

**Additional file 9: Figure S4.** ProMisE classification according to TMA and t-NGSdata.Distribution of molecular subtypes in our set.HRDMSIscore according to molecular subtype.TMBaccording to molecular subtype.Kaplan Meier curves for progression-free survival. MMRd: mismatch repair deficient; NSMP: non-specific molecular profile.

**Additional file 10: Figure S5.** Supervised analysis of copy number alterations comparing cases with or without disease progression. Dotted line: Threshold of significance associated with a False Discovery Rate < 0.25.

**Additional file 11: Materials S1.** aCGH data.

**Additional file 12: Materials S2.** Nanostring nCounter mRNA data.

## Data Availability

All genomic and transcriptomic data analyzed in this study are available in supplementary materials.

## Abbreviations

95 CI: 95% Confidence interval
CCC: Clear cell carcinomas
CNA: Copy-number-alterations
ER: Estrogen receptor
ESCAT: ESMO Scale for Clinical Actionability of molecular Targets
GISTIC: Genomic Identification of Significant Targets in Cancer
HR: Hazard ratio
HRD: Homologous recombination deficiency
ICI: Immune checkpoint inhibitors
ICR: Immunologic constant of rejection signature
IHC: Immunohistochemistry
MMRd: Mismatch repair deficiency
MSI: Micro-satellite instability
NSMP: Non-specific molecular profile
OS: Overall survival
OvCC: Ovarian clear cell cancer
PFS: Progression-free survival
PR: Progesterone receptor
ProMisE: Proactive Molecular Risk Classifier of Endometrial Cancer
SD: Standard deviation
TCGA: The Cancer Genome Atlas
TIS: T cell-inflamed signature
TMA: Tissue MicroArray
TMB: Tumor mutational burden
TMRG: Rare Gynecologic Malignant Tumors
t-NGS: Tumor next-generation sequencing
t-SNE: T-distributed Stochastic Neighbor Embedding
