# Supplementary figures and images for: Clinical, pathological, and comprehensive molecular analysis of the uterine clear cell carcinoma: a retrospective national study from TMRG and GINECO network
Source: J Transl Med. 2023 Jun 23;21:408. doi: 10.1186/s12967-023-04264-7 (PMC10288685; doi:10.1186/s12967-023-04264-7)

## Slide 1
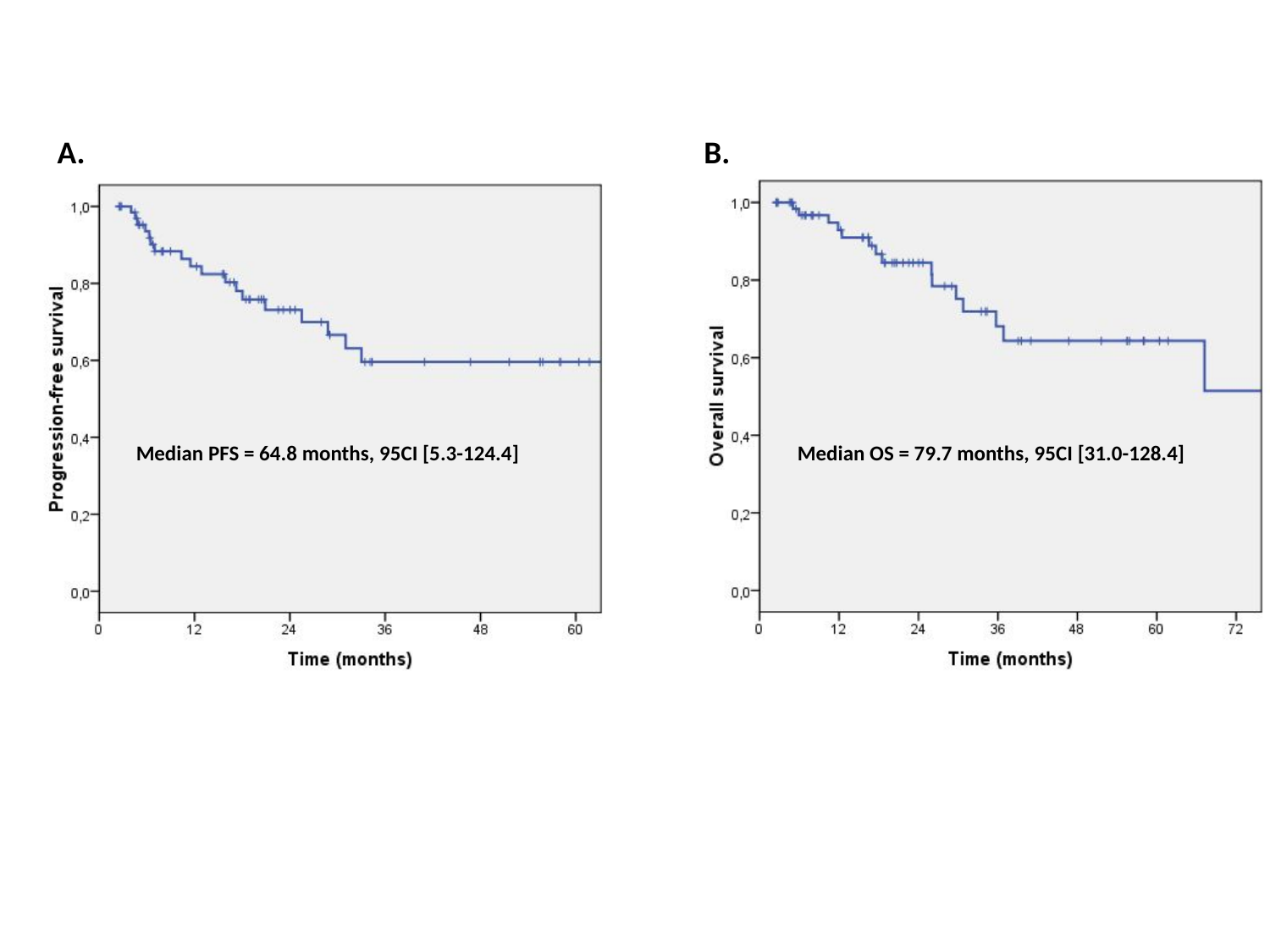

A.
B.
#
Median OS = 79.7 months, 95CI [31.0-128.4]
Median PFS = 64.8 months, 95CI [5.3-124.4]

Supplement: Supplementary file 6 — Additional file 6: Figure S1. Kaplan Meier curves for progression-free survivaland overall survival. [file 12967_2023_4264_MOESM6_ESM.pptx]

## Slide 1
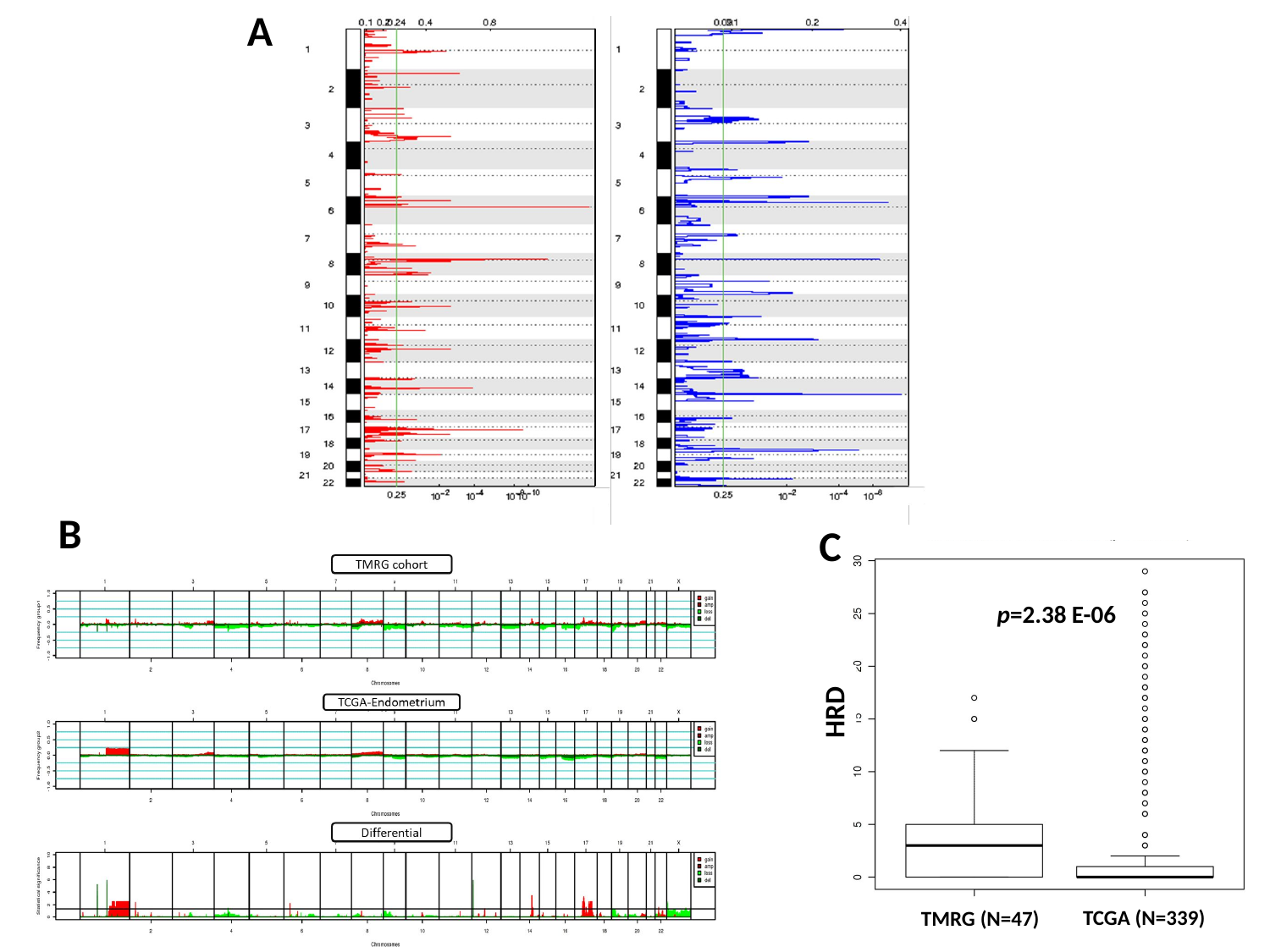

A
B
C
p=2.38 E-06
HRD
TCGA (N=339)
TMRG (N=47)

## Slide 2
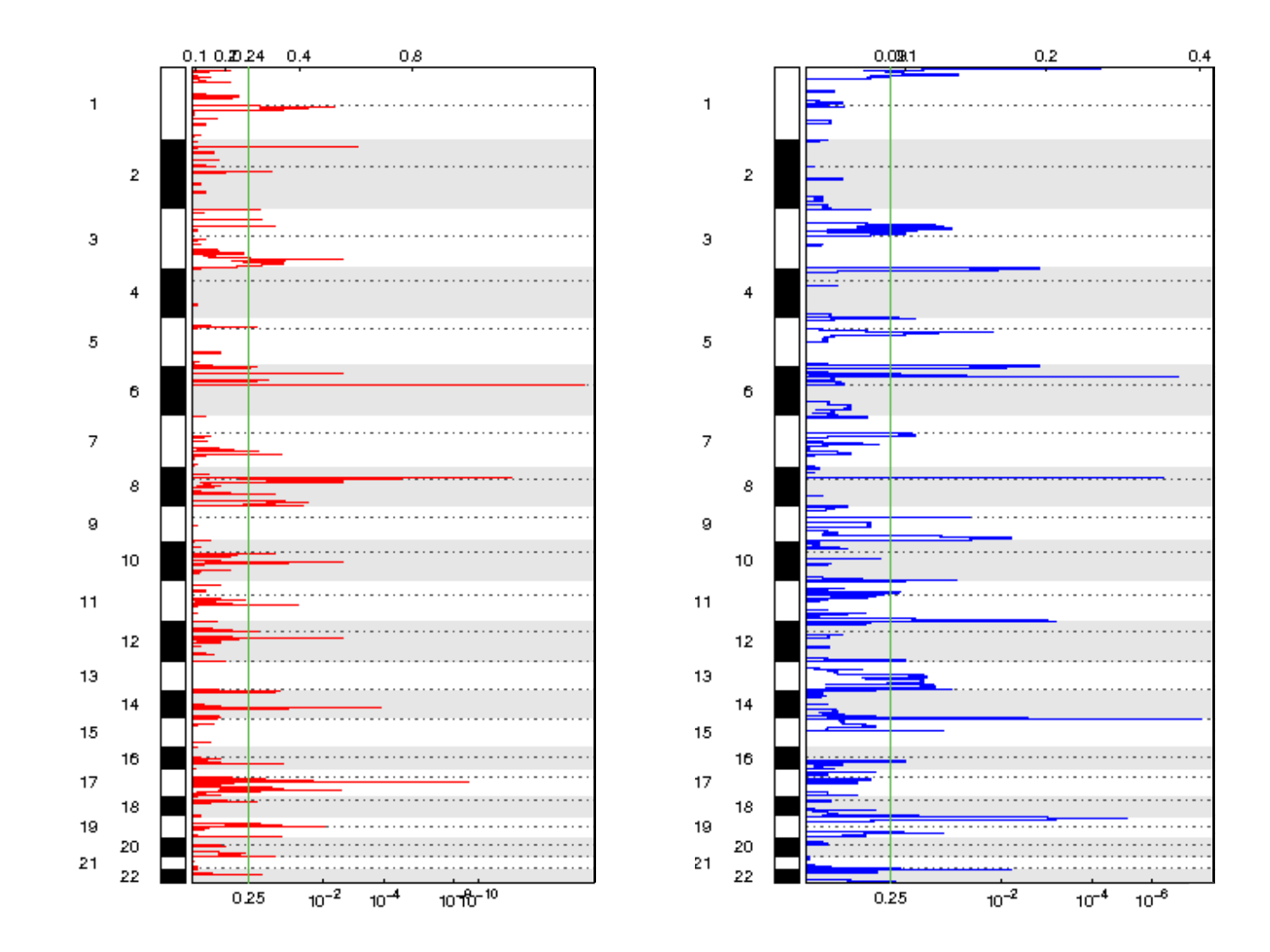

## Slide 3
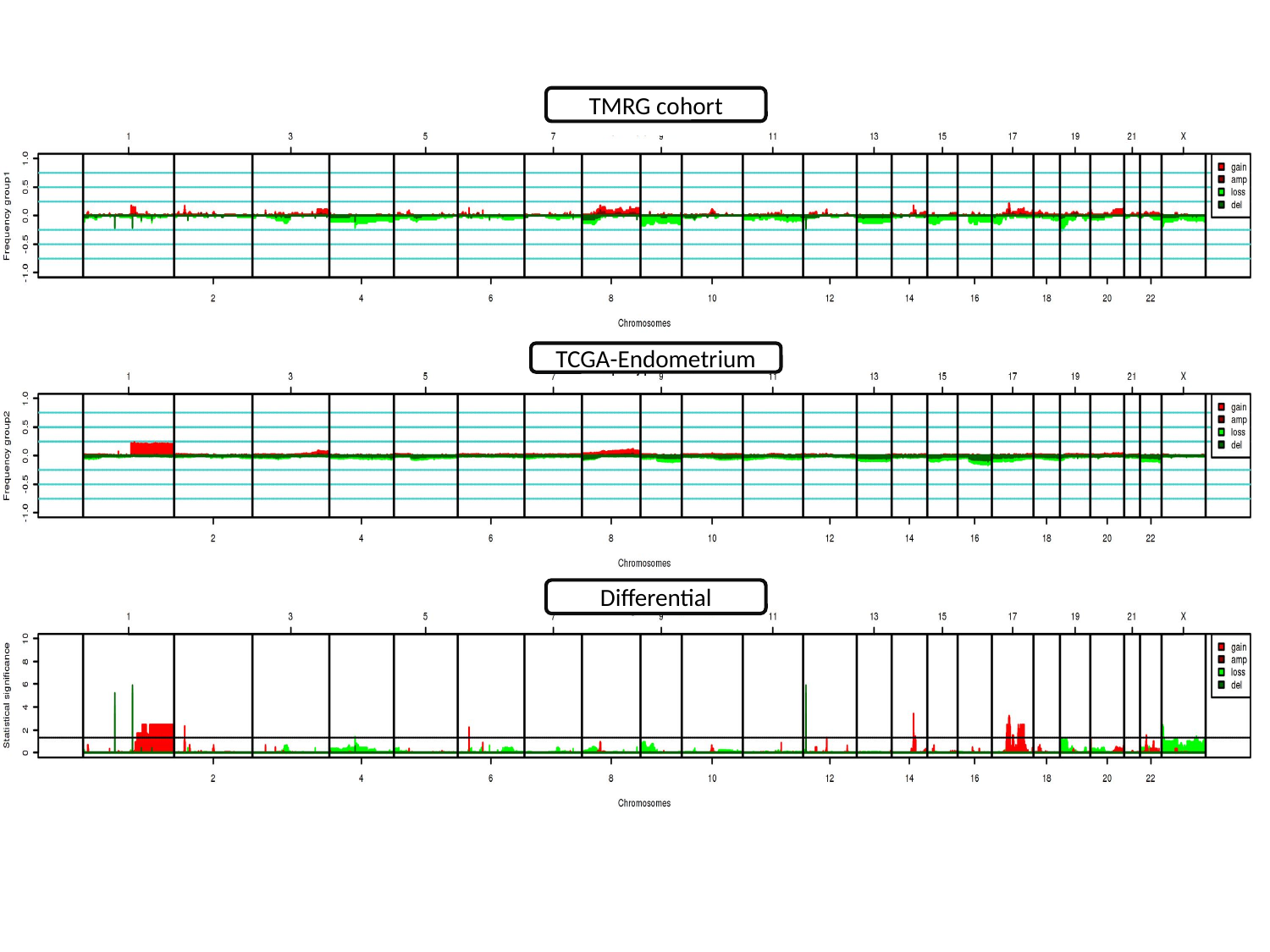

TMRG cohort
TCGA-Endometrium
Differential

Supplement: Supplementary file 8 — Additional file 8: Figure S3. Copy number alterations profiles of uterine CCC tumors from the TMRGcohort.Frequency plot of recurrent copy number alterations identified in UCCC tumors using the GISTIC algorithm. Frequencies of gainsand lossesare plotted as a result of chromosome location. X-axis: top = log–scale ratio; bottom = q-values. Green lines represent the threshold for significance.Supervised analysis comparing uterine CCC samples to endometrial tumors from TCGA dataset. Dotted line: Threshold of significance associated with a False Discovery Rate < 0.25.Homologous recombination deficiencyscore between TMRG and TCGA cohorts. [file 12967_2023_4264_MOESM8_ESM.pptx]

## Slide 1
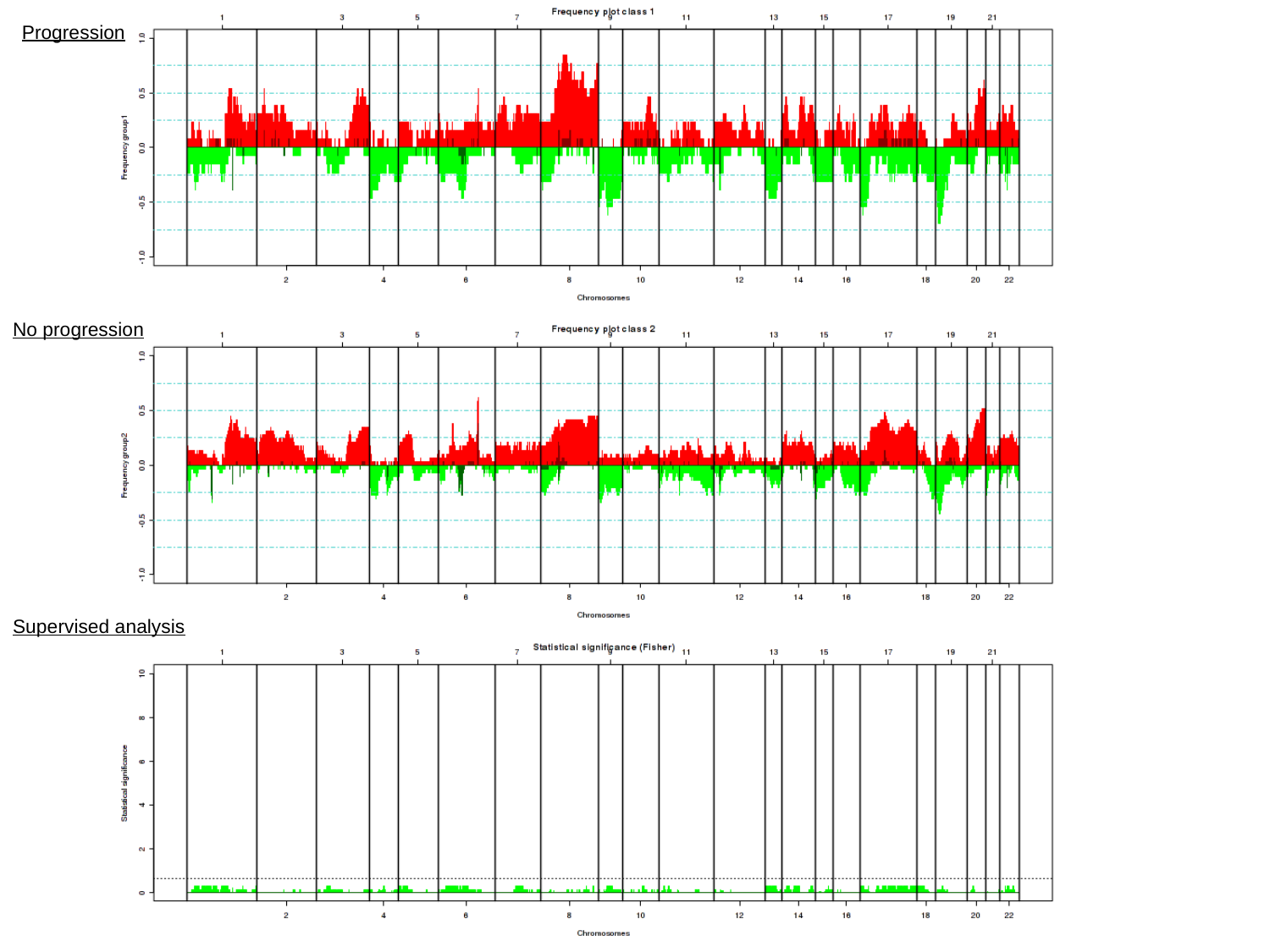

Progression
No progression
Supervised analysis

Supplement: Supplementary file 10 — Additional file 10: Figure S5. Supervised analysis of copy number alterations comparing cases with or without disease progression. Dotted line: Threshold of significance associated with a False Discovery Rate < 0.25. [file 12967_2023_4264_MOESM10_ESM.pptx]
