# Supplementary material for: Clinical, pathological, and comprehensive molecular analysis of the uterine clear cell carcinoma: a retrospective national study from TMRG and GINECO network
Source: J Transl Med. 2023 Jun 23;21:408. doi: 10.1186/s12967-023-04264-7 (PMC10288685; doi:10.1186/s12967-023-04264-7)
Supplement: Supplementary file 7 — Additional file 7: Figure S2. Supervised analysis of gene expression profiles of UCCC and a set of ovarian clear cells tumors.Volcano plots of differential mRNA expression between UCCC and ovarian clear cell tumorsand normal ovarian tissue. Filtering of ovarian tissue related genes allow idenitification of 207 differentially expressed genes. [file 12967_2023_4264_MOESM7_ESM.pptx]

## Slide 1
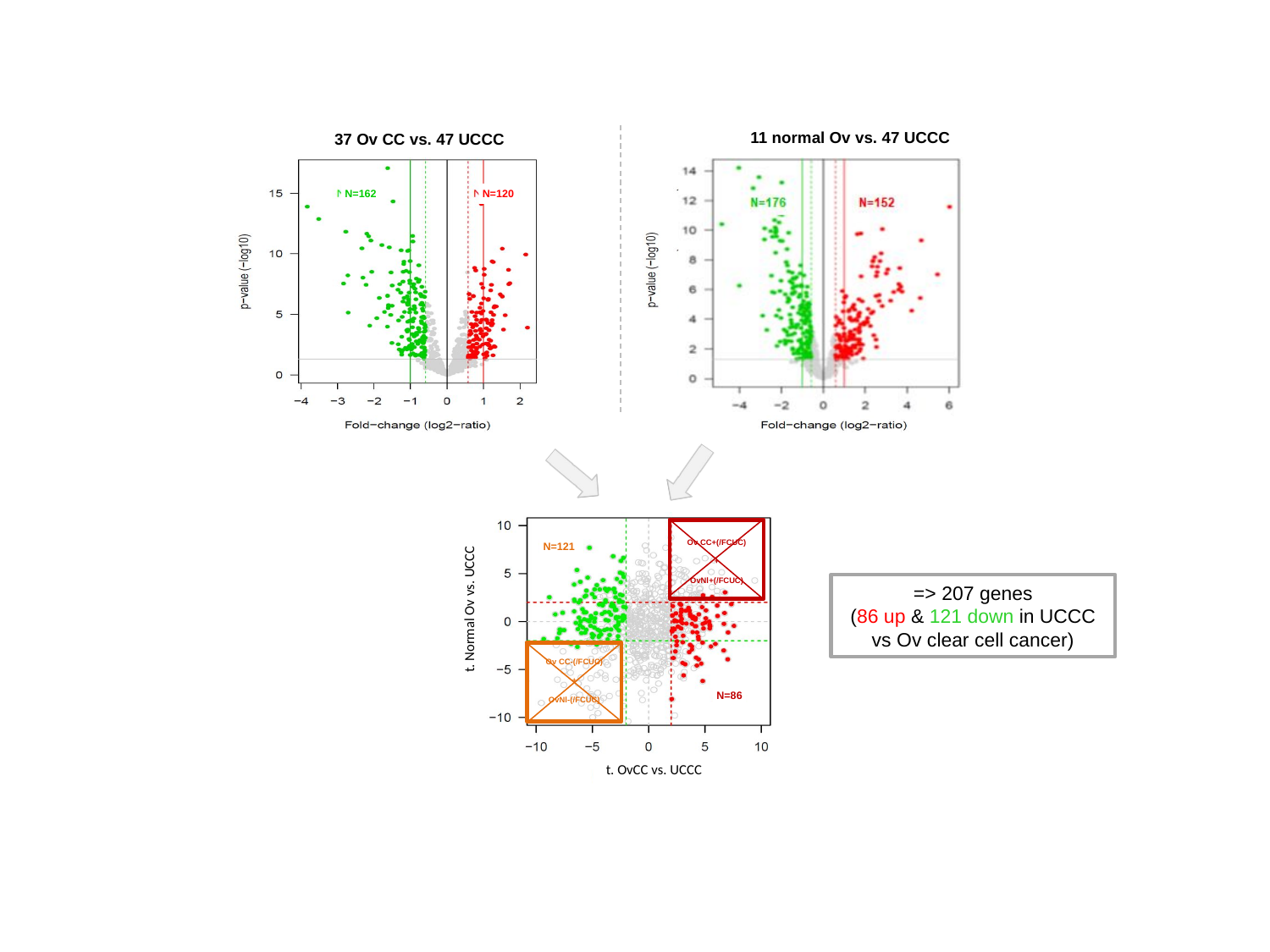

11 normal Ov vs. 47 UCCC
37 Ov CC vs. 47 UCCC
N=162
N=120
N=121
N=86
Ov CC+(/FCUC)
+
OvNl+(/FCUC)
Ov CC-(/FCUC)
+
OvNl-(/FCUC)
=> 207 genes
(86 up & 121 down in UCCC vs Ov clear cell cancer)
t. Normal Ov vs. UCCC
t. OvCC vs. UCCC

## Slide 2
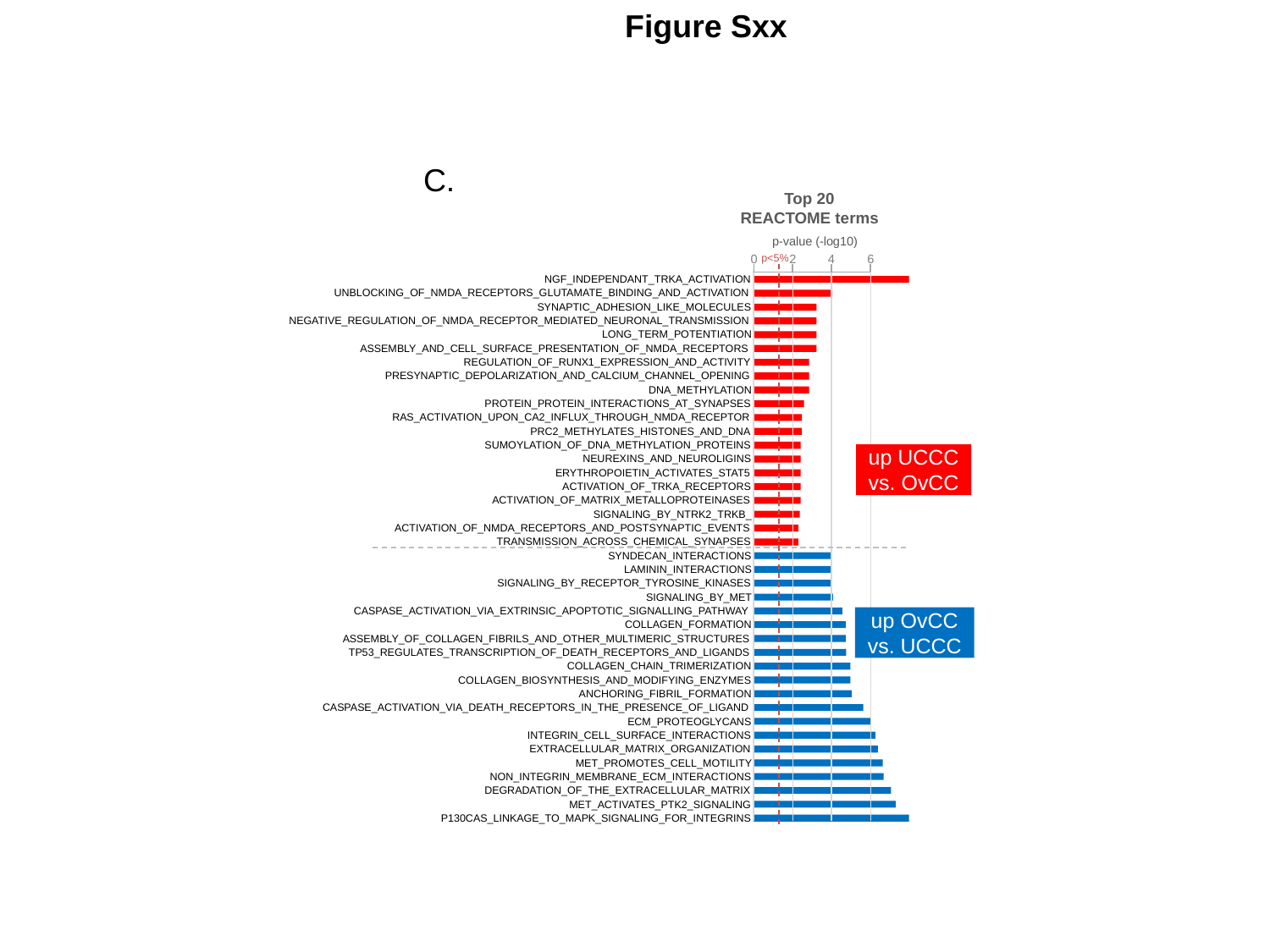

Figure Sxx
C.
Top 20
REACTOME terms
p-value (-log10)
0
2
4
6
p<5%
NGF_INDEPENDANT_TRKA_ACTIVATION
UNBLOCKING_OF_NMDA_RECEPTORS_GLUTAMATE_BINDING_AND_ACTIVATION
SYNAPTIC_ADHESION_LIKE_MOLECULES
NEGATIVE_REGULATION_OF_NMDA_RECEPTOR_MEDIATED_NEURONAL_TRANSMISSION
LONG_TERM_POTENTIATION
ASSEMBLY_AND_CELL_SURFACE_PRESENTATION_OF_NMDA_RECEPTORS
REGULATION_OF_RUNX1_EXPRESSION_AND_ACTIVITY
PRESYNAPTIC_DEPOLARIZATION_AND_CALCIUM_CHANNEL_OPENING
DNA_METHYLATION
PROTEIN_PROTEIN_INTERACTIONS_AT_SYNAPSES
RAS_ACTIVATION_UPON_CA2_INFLUX_THROUGH_NMDA_RECEPTOR
PRC2_METHYLATES_HISTONES_AND_DNA
SUMOYLATION_OF_DNA_METHYLATION_PROTEINS
NEUREXINS_AND_NEUROLIGINS
ERYTHROPOIETIN_ACTIVATES_STAT5
ACTIVATION_OF_TRKA_RECEPTORS
ACTIVATION_OF_MATRIX_METALLOPROTEINASES
SIGNALING_BY_NTRK2_TRKB_
ACTIVATION_OF_NMDA_RECEPTORS_AND_POSTSYNAPTIC_EVENTS
TRANSMISSION_ACROSS_CHEMICAL_SYNAPSES
SYNDECAN_INTERACTIONS
LAMININ_INTERACTIONS
SIGNALING_BY_RECEPTOR_TYROSINE_KINASES
SIGNALING_BY_MET
CASPASE_ACTIVATION_VIA_EXTRINSIC_APOPTOTIC_SIGNALLING_PATHWAY
COLLAGEN_FORMATION
ASSEMBLY_OF_COLLAGEN_FIBRILS_AND_OTHER_MULTIMERIC_STRUCTURES
TP53_REGULATES_TRANSCRIPTION_OF_DEATH_RECEPTORS_AND_LIGANDS
COLLAGEN_CHAIN_TRIMERIZATION
COLLAGEN_BIOSYNTHESIS_AND_MODIFYING_ENZYMES
ANCHORING_FIBRIL_FORMATION
CASPASE_ACTIVATION_VIA_DEATH_RECEPTORS_IN_THE_PRESENCE_OF_LIGAND
ECM_PROTEOGLYCANS
INTEGRIN_CELL_SURFACE_INTERACTIONS
EXTRACELLULAR_MATRIX_ORGANIZATION
MET_PROMOTES_CELL_MOTILITY
NON_INTEGRIN_MEMBRANE_ECM_INTERACTIONS
DEGRADATION_OF_THE_EXTRACELLULAR_MATRIX
MET_ACTIVATES_PTK2_SIGNALING
P130CAS_LINKAGE_TO_MAPK_SIGNALING_FOR_INTEGRINS
up UCCC
vs. OvCC
up OvCC
vs. UCCC
