# Supplementary material for: Clinical, pathological, and comprehensive molecular analysis of the uterine clear cell carcinoma: a retrospective national study from TMRG and GINECO network
Source: J Transl Med. 2023 Jun 23;21:408. doi: 10.1186/s12967-023-04264-7 (PMC10288685; doi:10.1186/s12967-023-04264-7)
Supplement: Supplementary file 9 — Additional file 9: Figure S4. ProMisE classification according to TMA and t-NGSdata.Distribution of molecular subtypes in our set.HRDMSIscore according to molecular subtype.TMBaccording to molecular subtype.Kaplan Meier curves for progression-free survival. MMRd: mismatch repair deficient; NSMP: non-specific molecular profile. [file 12967_2023_4264_MOESM9_ESM.pptx]

## Slide 1
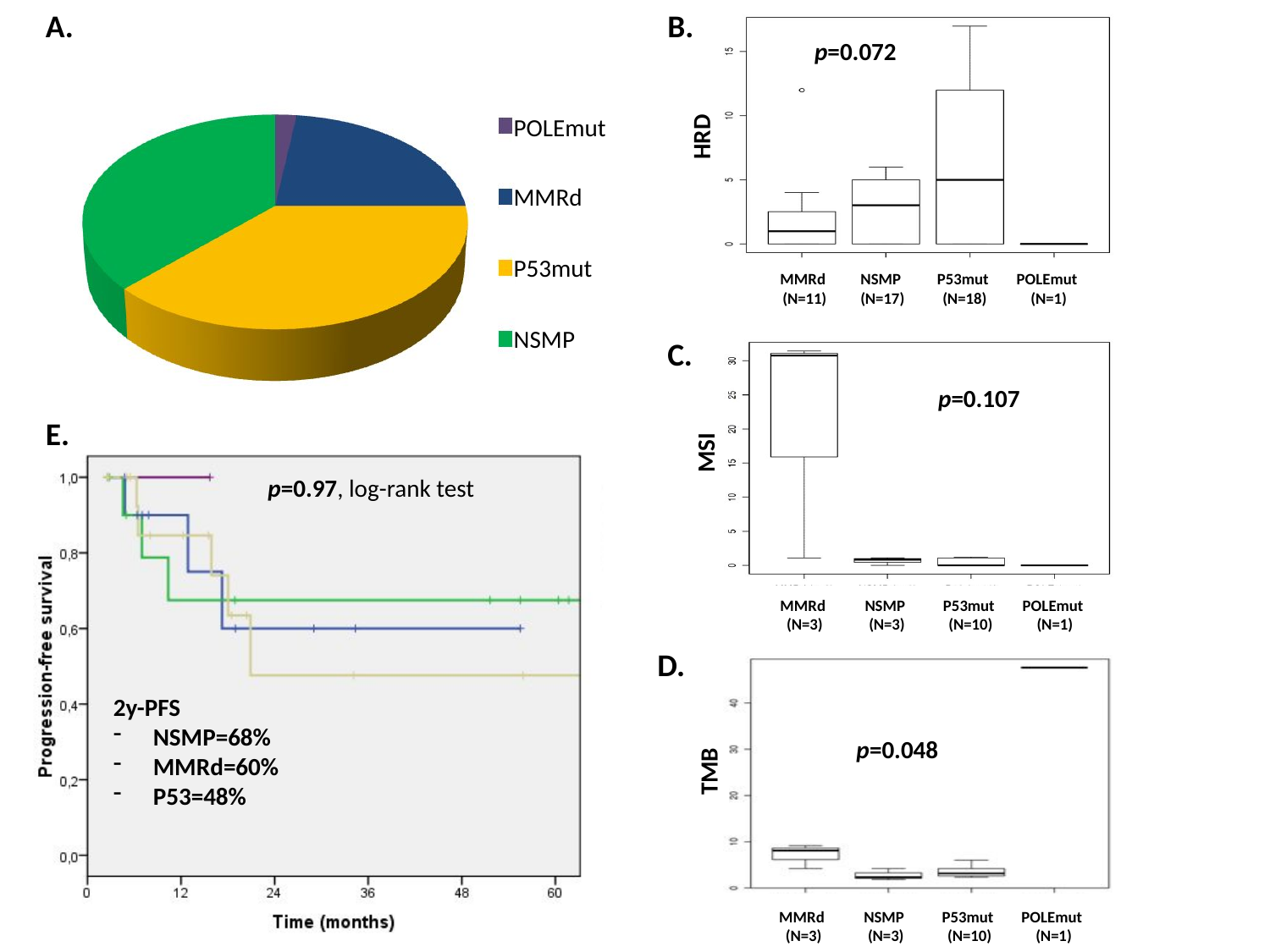

A.
B.
p=0.072
POLEmut
HRD
HRD
MMRd
P53mut
MMRd
(N=11)
NSMP
(N=17)
P53mut
(N=18)
POLEmut
(N=1)
MMRd
(N=3)
NSMP
(N=17)
P53mut
(N=18)
POLEmut
(N=1)
NSMP
C.
p=0.107
E.
MSI
p=0.97, log-rank test
MMRd
(N=3)
NSMP
(N=3)
P53mut
(N=10)
POLEmut
(N=1)
D.
2y-PFS
NSMP=68%
MMRd=60%
P53=48%
p=0.048
TMB
MMRd
(N=3)
NSMP
(N=3)
P53mut
(N=10)
POLEmut
(N=1)
